# The Willingness to Pay for Non-Alcoholic Beer: A Survey on the Sociodemographic Factors and Consumption Behavior of Italian Consumers

**DOI:** 10.3390/foods14132399

**Published:** 2025-07-07

**Authors:** Antonietta Baiano

**Affiliations:** Dipartimento di Scienze Agrarie, Alimenti, Risorse Naturali e Ingegneria (DAFNE), University of Foggia, 71122 Foggia, Italy; antonietta.baiano@unifg.it; Tel.: +39-(0)881338149

**Keywords:** alcohol-free, consumption behavior, fermented beverages, habits, Italian market, sociodemographic factors

## Abstract

The Italian market for non-alcoholic beer is very small, with a volume per capita of around 0.7 L. However, there are interesting prospects for future growth for reasons ranging from strict traffic code rules on the quantity of alcohol ingested to simple curiosity. This research aimed to investigate the willingness of Italian consumers/potential consumers to pay for non-alcoholic beer. To accomplish this, a questionnaire was administered using the Google Forms application. Three hundred and ninety-two people participated in this survey voluntarily and without monetary compensation. A probit regression model was used to estimate the impact of certain sociodemographic characteristics (number of inhabitants of the place of residence, region of residence, age group, gender, education level, employment situation, and annual net income), participants’ consumption habits with respect to alcoholic beer, and participants’ knowledge of and preference for non-alcoholic beers with respect to willingness to pay for non-alcoholic beers. The prices respondents were willing to pay ranged from EUR 1.51 to 2.00 for a 33 cL glass bottle. Only two factors significantly affected (*p* < 0.1) non-alcoholic beer WTP, namely, “Age” and “Non-alcoholic beer color”. WTP decreased as the age of the respondents increased and was higher for the darker beer.

## 1. Introduction

In recent years, researchers, companies, and consumers have expressed curiosity and interest regarding low-alcohol and alcohol-free versions of conventional alcoholic beverages [1]. With this growth in interest, terms such as “no”, “free”, “zero”, “low”, “light”, and “reduced” have proliferated; in a globalized market, where each country or union of countries has specific regulations, these terms can generate confusion among consumers regarding real alcohol content and increase the risk of producers incurring sanctions for using terminology that does not accord with current legislation. Therefore, it is desirable to establish a common commercial nomenclature at the Codex Alimentarius level [1].

To understand the real dimensions of this phenomenon, it is appropriate to consider the size of the market for these types of beers. In 2023, 2.39 billion liters of non-alcoholic beer were sold worldwide [2]. In 2019, according to Kokole et al. [3], approximately 1.38 billion liters of non-alcoholic beer (ABV ≤ 0.5%) were produced and sold in EU-27 countries, i.e., with the UK included (3.8% of the total beer volume produced in the EU). The market leaders were Germany (30.5%), Spain (16.8%), the Netherlands (14.4%), Poland (12.0%), and Czechia (7.1%). In the same year, more than 200 million liters of non-alcoholic beer were exported outside of EU-27 countries, and around 10.4 million liters were imported, with an apparent consumption of 1,003,281,706 L (3.1% of the beer consumed in the EU), equivalent to 2.2 L per capita. In the UK and the Netherlands, two of the countries that lead the ranking of no- and low-alcohol beer consumption, the typical drinker of these beers is young, better educated, and wealthier [4,5]. According to data from Statista [6], the sales of non-alcoholic beer in Italy had a market share of 1.8% in 2022, and the volume per capita was around 0.7 L, consumed mainly at home. This data places Italy among the countries in which this phenomenon must be followed carefully to understand what draws consumers to NoLo beer.

Low-alcohol beers can be produced in different beer styles. However, based on a word frequency analysis (word cloud) performed on US brewery websites that joined beer style to beer alcohol content, Myles et al. [7] found that low alcohol contents were more frequently combined with terms such as “ale”, “lager”, “blonde”, “IPA”, and “pilsner”. Specifically, ales accounted for 62% of the low(er)-alcohol beers identified.

According to Waehning and Wells [8], the list of factors impacting the consumption of NoLo beverages includes the following: product extrinsic factors (labeling, branding, availability, packaging, and price); product intrinsic factors (product sensory characteristics); individual biological/physiological factors (gender, age, genetics, and whether one is a traditional alcohol beverage drinker); individual psychological factors (motives, involvement, emotional responses, subjective norms, purchase experience, and novelty seeking); environmental situational factors (pairing with foods or not, time of day, and drinking occasion and place); and environmental sociocultural factors (education, income, religion, and social grade). In a study carried out in Nova Scotia (Canada), Moss et al. [9] identified people most willing to drink NoLo beer as those interested in a healthy lifestyle and those who want to reduce their alcohol consumption. However, negative expectations of the taste of NoLo beer are the main limits preventing the consumption of this type of beer. Furthermore, labeling beer as alcohol-free seems sufficient to reduce consumer interest [10].

A scoping review of English-language articles published between 2010 and 2021 and indexed in PubMed and Web of Science highlighted that the key drivers of the purchase of NoLo beers include taste, prior experiences, brand, health issues, price differentials, and a decrease in the social stigma associated with drinking NoLo beverages [11].

Day et al. [12] investigated the willingness to purchase and pay for non-alcoholic beers among Australian consumers. First, they observed that prior experience with non-alcoholic versions positively influenced intention to consume and WTP. Furthermore, their respondents declared themselves willing to pay slightly less for non-alcoholic beer compared with its alcoholic counterpart and, in any case, substantially less than the average market price for the non-alcoholic version. The only study that investigated Italian consumers’ attitude and WTP for non-alcoholic beer is from 2008 [13]. By applying a quantitative concept analysis, the authors individuated the key factors in the following decreasing order: packaging, with glass and twist-off caps increasing consumer preference and plastic decreasing it; pricing, with prices lower than EUR 0.80 increased NoLo preference, while prices higher than EUR 1.25 exerted the greatest negative impact on preference; and sensory characteristics, such as malty and fruity flavors and a remarkable body, which exerted positive impacts on consumer preference, while the use of organic raw materials negatively affected consumer preference. Since this study, the trend of non-alcoholic beer consumption in Italy has significantly changed, with the sales of non-alcoholic beer generally having decreased, going from a market share of 2.6% in 2014 to the already-cited 1.8% in 2022. At the same time, the surrounding conditions and sociodemographic characteristics of the Italian population have also changed, making a re-evaluation necessary. The present research aimed to investigate interest in and willingness to purchase/pay for NoLo beer among Italian consumers/potential consumers. The influence of factors such as place of residence, age, gender, education level, occupation, income, and consumption habits was evaluated.

## 2. Materials and Methods

### 2.1. Questionnaire Structure

The questionnaire was structured into the following sections: The first section contained a brief description of the purposes of the survey; an invitation to collaborate in the research by answering questions; and finally, a statement on data collection, carried out exclusively for statistical purposes and in an anonymous way. The second section included the following 7 sociodemographic questions: number of inhabitants of the place of residence; region of residence; age group; gender; education level; employment situation; and annual net income. The third section consisted of 8 questions regarding consumption habits for alcoholic beer; knowledge and preference for non-alcoholic beers; and willingness to pay (Table S1).

### 2.2. Questionnaire Administration

The questionnaire was administered using the Google Forms application. The link to the questionnaire was publicized on messaging platforms. Once the questionnaire was completed, the respondents had the following options: to review the answers previously provided by clicking on the “back” button; to send their answers definitively by clicking on “send”; or to delete the completed questionnaire by selecting the “delete form” option. The methodological rigor of the research was guaranteed because the procedure was previously tested in three pilot studies on craft beer and other low-alcohol beverages among Italian consumers. Those studies quantified the response rate, ranging between 2.2% and 2.9% of the questionnaires administered, and the minimum number of people needed to respond to the questionnaire (at least 15,909) to guarantee at least 350 completed questionnaires. To guarantee a certain safety margin, 17,000 questionnaires were sent, obtaining 392 responses (response rate, 2.3%). A stratification technique was applied by sending the questionnaire proportionally to the population of each Italian region; distributing the population by age groups; and distributing by gender [14].

People participated in the survey voluntarily and without monetary compensation. The minimum age required to participate was 18 years old. The questionnaire included a statement on data collection stating that it was carried out exclusively for statistical purposes and in an anonymous way, and informed consent was assumed when the participants sent their answers definitively by clicking on “send”. An appropriate protocol for protecting the rights and privacy of all participants was utilized during the execution of this research. The questionnaire concerned knowledge, preferences, and consumption habits. It did not include any product tasting. In Italy, the legislation governing Ethics Committees and clinical trials (for example, Legislative Decree 211/03 [15]) allows for exemptions from ethical approval for studies with negligible risk to participants that do not involve invasive or potentially harmful procedures. Due to all the statements listed above and the cited legislation, ethical approval was not required.

### 2.3. Statistical Analysis

First, a descriptive analysis of the sociodemographic characteristics, product knowledge, and consumption habits of the survey participants was carried out, and the average WTP was determined. These data were also subjected to ANOVA (*p* < 0.05) to determine statistically significant differences in WTP across the various levels of each factor, and regression analysis (*p* < 0.05) was used to evaluate the relationship between each factor and WTP. Then, a probit regression model was applied to estimate the impact of the factors on the prices that respondents were willing to pay. Statistical analyses were carried out using Statistica for Windows V. 7.0. (Statsoft, Tulsa, OK, USA) at *p* < 0.1.

## 3. Results and Discussion

### 3.1. Sociodemographic Categories of Respondents

The distribution of the respondents by sociodemographic categories is reported in Table 1. About half of the respondents lived in medium-sized Italian cities, and the majority resided in Southern Italy. This result was of particular interest, especially considering that the questionnaire was administered in various Italian regions in proportion to the resident population. According to Knibbe et al. [16], the rate of beer drinkers and beer consumption is lower in Mediterranean regions located near northern European countries and higher in areas farther from them. Although the ANOVA did not reveal statistically significant differences in the average WTP (EUR 2.25–2.73 per 33 cL pack) between locations with different numbers of inhabitants, differences in the maximum price were recorded. The WTP values were recorded up to EUR 4.00 in locations where the number of inhabitants did not exceed 20,000; EUR 4.50 in cities with more than 500,000 inhabitants; and up to EUR 5.00 in towns with an intermediate number of inhabitants. In small municipalities, traditional alcoholic beer might play a more central role in social gatherings, thus lowering consumers’ willingness to pay for NoLo beers, while the ample availability of other non-alcoholic alternatives may lower the WTP for low-alcohol beer in larger cities. This study highlights significant differences in the average WTP between Italian regions, with consumers from Basilicata and Toscana, respectively, declaring the lowest and highest average willingness to pay (EUR 1.50 and EUR 4.00 per 33 cL pack). Differences among regions were also highlighted for the maximum WTP, which was between EUR 1.00 and EUR 1.50 for respondents from Basilicata and EUR 4.51–EUR 5.00 for respondents from Puglia and Emilia-Romagna. There is no reference in the literature that can explain the reduced willingness to pay for alcohol-free beer observed in Basilicata. However, it could be linked to a greater propensity to purchase traditional foods and beverages by Lucanian consumers.

Among respondents, WTP for non-alcoholic beer was in the following decreasing order across the age ranges: 51–60 years old; 41–50 years old; and finally 18–30 years old. Although it is generally assumed that young people are more willing to try new types of drinks, our results, as well as those of Knibbe et al. [16], only partially support this assumption. This anomalous distribution of age groups in our survey can be explained by considering that ‘youth’ is not always synonymous with ‘greater willingness’ to experiment with new products. Furthermore, consumers between 40 and 60 years old might have been motivated to participate in the survey not only out of curiosity but also due to a greater willingness to adopt healthier lifestyles and food consumption following the onset of age-related diseases [17]. Differences in the maximum price were recorded across the age groups. The maximum WTP values were between EUR 3.51 and EUR 4.00 for respondents belonging to the 31–40 (Gen Y or Millennials) and 71–80 (Baby Boomers) age groups. Among the remaining respondents (Gen X and Gen Z), the maximum WTP reached values between EUR 4.51 and 5.00. Although the consumption trend for NoLo drinks is driven by both Gen Z and Gen Y [18], the present study highlights that the two age groups ascribe different economic values to these products, probably because, in Italy, Gen Y is already in the workforce and—by attributing value to products based on perceived income—is more inclined to save money than Gen Z, who are still financially dependent on their parents (often Baby Boomers). The lowest WTP declared by Baby Boomers relative to Gen X is probably a consequence of the fact that they are approaching retirement or, even though they are still working, have to dedicate their economic resources to helping their children.

The survey participants were almost equally distributed between male and female genders, with a slight preponderance of the latter. ANOVA analysis highlighted a statistically significant difference between genders, with a higher average WTP for women than men (EUR 2.64 vs. EUR 2.29). This result indicates a different situation in the Italian market compared with the Finnish one, where men purchase non-alcoholic beer more often than women [19]. Conversely, in Germany, NoLo drink consumption is generally associated with health-conscious women [20]. Instead, no significant difference was highlighted for the maximum WTP values between men and women. According to the regression analysis, gender was the only sociodemographic factor with a significant but weak (*p* = 0.011, R = 0.185) relationship with WTP, meaning that gender could predicate willingness to pay for this type of beverage.

Only 5.8% of respondents declared a low education level, consistent with the finding that interest in non-alcoholic beer is most common among highly educated consumers [19]. Consistent with this finding, respondents with a compulsory education level were willing to pay a lower maximum price (EUR 3.51–4.00) than respondents with higher education levels (EUR 4.51–5.00). No statistical difference was observed between the different cultural levels for the average WTP (EUR 2.38–2.53). This means that although awareness of the value of non-alcoholic beer is considerable across the population, a higher cultural level increases the willingness to pay for this type of product, marking a certain level of segmentation among NoLo beer consumers [21].

Around one-third of respondents worked as employees and one-fifth were students. The other work categories were poorly represented. The annual net income declared by the respondents predominantly fell in the following ranges: EUR 18,001–24,000; lower than EUR 12,000; and EUR 24,001–30,000. Our respondents had higher net incomes than the overall distribution of Italian taxpayers [22]. According to the ANOVA results, the average price that respondents in the various employment categories were willing to pay differed significantly. Specifically, the lowest average (EUR 1.83) and maximum WTP values (EUR 2.51–3.00) were declared by housewives. The highest average WTP values were expressed by managers (EUR 2.93), followed by unemployed and retired people (EUR 2.82–2.83). Just as surprisingly, the highest maximum WTP values (EUR 4.51–5.00) were registered not only among managers but also among students, employees, and unemployed people. Equally surprising are the binomial data relating income and willingness to pay. No difference was observed in terms of the average WTP value of (EUR 2.31–2.82). The maximum price respondents declared that they were willing to pay was totally unrelated to income: between EUR 3.51 and 4.00 for incomes ranging from EUR 12,000 to 18,000 and EUR 36,001 to 50,000; EUR 4.01 and 4.50 for incomes ranging from EUR 18,001 to 24,000 and EUR 30,001 to 36,000; and EUR 4.51 and 5.00 for incomes below EUR 12,000, between EUR 24,001 and 30,000, or above EUR 50,000. It is plausible that willingness to pay does not necessarily translate into an actual purchase. However, the data obtained from this survey opens up further areas of investigation because, in the Italian market, the behavior observed by Anderson et al. [4] in a survey of British consumers does not apply (i.e., a greater propensity among more affluent and educated people to choose products with low or zero alcohol content).

### 3.2. Distribution of Total Respondents by Consumption Habits for Alcoholic Beer, Knowledge and Preference for Non-Alcoholic Beers, and Willingness to Pay

The corresponding data are reported in Table 2. Regarding the first question on consumption habits for alcoholic beer, the respondents declared that they consume alcoholic beer almost everywhere, without a particular preference between public and private places. Indeed, beer is the typical drink of the masses, and its low prices (at least with reference to industrial beer) do not represent an obstacle to its consumption outside the home. The ANOVA highlighted that the place of beer consumption did not significantly affect the average WTP of non-alcoholic beer, which was between EUR 2.42 and EUR 2.52. Instead, the place of consumption significantly influenced the maximum price that respondents said they would pay, which was lower for those accustomed to consuming beer in pizzerias. This finding is explained by the fact that a pizzeria is considered a place where it is possible to have dinner without spending a lot of money, given the low cost of the pizza–beer combination. It is interesting to highlight that clubs did not represent the preferred place of consumption for any of the respondents.

Most consumers of alcoholic beer declared a strong preference for blonde beers, in line with the findings of De Pascale et al. [23] and Cascone et al. [24], who analyzed the preference of Italians for craft beers. This result is explained by the segmentation of the Italian beer market, with blond lagers representing 82,73% of the total volume, knowing that a high level of familiarity positively shapes consumer preference [25,26]. Dark beer was the least favorite of all, probably because of inexperience with this type of beer [27]. However, the ANOVA highlighted a significantly higher average WTP for blanche and red beers (EUR 2.93 and 2.69, respectively) and a significantly lower WTP (EUR 2.33) just for blonde beers. The reason is that, in the viewpoint of most Italian consumers, lager beer is the classic thirst-quenching drink to be consumed very cold on the beach, almost like water, and, therefore, cannot have a price that is too high. If we consider the maximum price that respondents are willing to pay based on the color of the beer, the preference of Italians for light beers is clearly highlighted, with a maximum WTP value in a range of EUR 4.51–EUR 5.00 compared with EUR 4.01–EUR 4.50 for dark beers. An even more marked preference for pale beers was expressed in the case of non-alcoholic beers.

Regarding practical knowledge of alcohol-free beer, more than 63% of respondents said they had drunk it, and around 24% were curious to taste it even though they had not done so previously. This is quite a surprising answer, as a previous study by Silva et al. [28] on how Dutch and Portuguese people conceptualize non-alcoholic beer consumption found that it triggers neutral or negative emotions, specifically rational, conscious, or disappointed. Moreover, NoLo beers are generally not perceived as hedonic beverages but only as functional substitutes for alcoholic beers [28]. According to the ANOVA, the average WTP was not significantly affected by previous tasting experiences (EUR 2.42–2.67). Instead, the maximum price that respondents said they were willing to pay for NoLo beer was lower among those who, having never drunk it, were planning to try it (EUR 4.01–4.50). This distrust or caution not only reflected a lack of knowledge of the products but also a lack of consensus regarding their pleasantness and health-related effects [29].

Regarding the reasons why respondents drank or might decide to drink non-alcoholic beer, they mainly indicated curiosity and the possibility of getting behind the wheel without problems. The reasons for drinking non-alcoholic beer did not influence the mean WTP but did affect the maximum WTP, which was higher (EUR 4.51–5.00) among those driven by curiosity, the ability to drive without problems, and health reasons. The options related to the effects on health, the calories introduced, and the possibility of choosing between multiple alternatives were rarely chosen. This is another surprising result, as Ramírez Pagès et al. [30] indicated that consumer concerns about health and prevention of the negative effects of alcohol were the primary motivations for purchasing NoLo drinks, particularly among people over 30 years old.

Packaging format is a product selection criterion that stimulates the senses of sight and touch [31]. The survey respondents identified 33 cL glass bottles as the preferred format for purchasing non-alcoholic beer. Concerning packaging, a previous study by Nieto-Villegas et al. [32] highlighted that beer consumers with a better attitude toward sustainability value glass bottles over cans. However, not all consumers have sustainability among their prevailing interests. According to Luís [33], consumers who want to reduce their alcohol consumption tend to prefer smaller formats, but this finding does not explain the choice of the 33 cL format for NoLo beers. Van Doosselaere [31] found that the 33 cL bottle is unanimously the favored packaging format in each context, followed (in decreasing order) by the 25 cL bottle, the 75 cL bottle, the 33 cL can, and the 50 cL can, the last of which also being the least popular in the present study. Perhaps the glass bottle is the most commonly chosen receptacle because it is considered an indicator of the quality of the product contained within, while the 33 cL format is the most preferred format because it is easy to drink on its own (in our study, the 25 cL format was not considered since it is uncommon in Italy). Consistent with the high quality ascribed to both glass bottles and the beverages contained within them, the ANOVA highlighted significant differences between the various packaging formats in terms of the maximum price that respondents were willing to pay: it was the highest for 33 and 50 cL glass bottles (EUR 4.51–5.00) and the lowest for 50 cL cans (EUR 2.51–3.00) (these prices were normalized by beer volume to be comparable).

Over 56% of respondents declared that, in a public place, they prefer to consume non-alcoholic beer on tap, probably because of the impression that draft beer is fresher than bottled beer, often tasting closer to that just brewed, whereas bottled and canned beers have undergone further processing. However, the ANOVA did not reveal significant differences between the two service modes.

Finally, concerning the respondents’ willingness to pay, the range corresponding to the apex of the WTP’s Gaussian distribution was between EUR 1.51 and 2.00 for a 33 cL bottle, a price greater than or equal to the average price of the most common alcoholic beers sold online [34]. It is also higher than the price for which Porretta and Donadini [13] found positive utility values for 66 cL glass bottles, with prices lower than EUR 1.00. A study performed on the Australian market highlighted that most respondents were willing to pay AUD 3.99 (the equivalent of EUR 2.29) or less, less than the average price for the alcoholic version [12].

### 3.3. Prediction of Willingness to Pay for Non-Alcoholic Beers

The list of possible WTP predictors for non-alcoholic beers on the Italian market is reported in Table 3. However, based on the *p*-values, only two factors significantly affected (*p* < 0.1) the respondents’ willingness to pay: age and the type of non-alcoholic beer (i.e., the color of the non-alcoholic beer) that the respondents preferred or would prefer to drink. Notably, the values of the standard error were quite high, highlighting the high variability of the data collected.

According to the negative sign of the corresponding coefficient, the willingness to pay for non-alcoholic beers decreased as the age of the respondents increased, probably due to the greater experience of older respondents with alcoholic beers. This assumption is supported by the findings of Mintel et al. [35] and Anderson et al. [4]. The former observed that NoLo drinks are particularly promising in younger markets with 64% of consumers aged between 18 and 24. The latter found that buying non-alcoholic beer is much more likely to occur among younger people.

The significance of color is probably related to the classification of beers based on that factor and to the indisputable fact that color often represents the only (surely the first) criterion used by consumers when selecting beer to buy [36]. Surprisingly, WTP was higher for darker beer, even though respondents gave the lowest percentage of preferences for this type of beer. This result agreed with the findings of Reinoso-Carvalho et al. [37], who found that survey participants were willing to pay up to 6% more for dark beer than for pale ones. However, in that study, most participants reported their preference for pale beers prior to tasting and expressed their WTP only after tasting both beers, meaning that the flavor of the dark beer positively influenced the hedonic experience of the consumers. In the present study, respondents were willing to pay more for a dark non-alcoholic beer even though they preferred the blond ones and had not tasted the beers themselves. This seemingly contradictory result can be explained by considering that, before consumption, color drives consumer expectations about the sensory and hedonic characteristics of food and beverages. Regarding beer, prior to or in the absence of tasting, people expect dark beer to be more bitter and stronger and, therefore, less pleasant than pale beer. However, the fuller body and flavor complexity of dark beers are considered as resulting from combinations of ingredients and brewing processes that are more complex and, consequently, more expensive than those employed in the production of lager beers. This indirect association between beer color and cost means that, recognizing a higher production cost for dark beer, potential consumers are willing to pay more despite its lower appeal [38,39].

Among Italian consumers, social factors (such as place of residence, gender, education level, and income), previous tasting experience with non-alcoholic products, consumer habits, and non-alcoholic beer packaging did not significantly affect their WTP. There may be several reasons for this finding. The first reason could be related to a lack of knowledge about alcohol-free beers among the various segments of the Italian population, which was counteracted solely by the greater propensity of the younger population to experiment with new products. Another reason could be linked to the still low diffusion of NoLo beers in the Italian market; the greater availability of these products would be a useful tool to reduce alcohol consumption, at least among the most socially advantaged segments of society, and to increase the WTP for alcohol-free alternatives [4]. Finally, one cannot ignore that alcohol consumption patterns in Italy are constantly evolving, preventing researchers from precisely identifying the significant socio-economic factors influencing the consumption of and/or WTP for NoLo drinks and quantifying their impacts in this country. In fact, according to a report by the Italian Minister of Health, from 2012 to 2022, changes in alcohol consumption habits were widespread in all age groups, highlighting a significant increase in consumers of alcoholic beverages (from 64.6% to 67.1%), a generalized decline in daily consumption, and (at the same time) increases in occasional and out-of-meal consumption and binge drinking. Furthermore, in this decade, the share of female consumers significantly increased for occasional (from 39.3% in 2012 to 46.9% in 2022) and out-of-meal consumption (from 15.6% to 23.2%) [40].

A deeper analysis of the probit regression model was necessary to evaluate model fit. The chi-square test provided a *p*-value equal to 0.3368205, indicating the limited significance of the relationship tested, a consequence of the already cited variability of the data. This result is also influenced heavily by the poor knowledge of alcohol-free beer among the respondents, as well as a certain distrust regarding the goodness of its organoleptic characteristics, which is widespread among Italian consumers. This state of affairs suggests that the results would change in the presence of a reversal of the trend in current consumer habits; an increase in the number of people interested in a healthy lifestyle; and an increase in NoLo beer knowledge among Italian consumers, which could modify the answers to the questionnaire as it involves conscious effort and careful consideration of information. Furthermore, a possible reason for the weak explanatory power of the regression model could be the omission of other factors that are potentially influential on consumer willingness to pay.

Although limited to the Italian population (the main limitation of this study), the approach adopted and the results obtained could be applicable to all countries, especially in the European Union, where beer and other alcoholic beverages are traditionally consumed and where the diffusion of such beverages in the alcohol-free version is still very low [3].

## 4. Conclusions

To understand the factors influencing the price Italian consumers are willing to pay for non-alcoholic beers, this study conducted a survey of sociodemographic factors, previous tasting experience with non-alcoholic products, consumer habits, and packaging/format. All of these factors can influence consumer preferences in a market where the diffusion of non-alcoholic beer is still limited, and the results highlight interesting growth prospects.

Regarding the potential Italian market for alcohol-free beer, the statistical processing of the data collected with this survey through ANOVA and simple regression analysis highlighted trends that often differ from those documented in the literature for markets in other countries. It also highlighted the rather complex relationships between sociodemographic factors/consumption habits and WTP. The respondents were willing to pay an average price of EUR 1.51–2.00 for a 33 cL glass bottle of non-alcoholic beer. That price is higher than the price of most commercial alcoholic beers that can be purchased on websites. Surprisingly, age and the color of non-alcoholic beer were the only factors that significantly affected (*p* < 0.1) the respondents’ willingness to pay, according to our probit regression model. The still poor knowledge of such products among Italian consumers and the aforementioned complex relationships may underlie the lack of a significant influence of these factors in the model, including the region of residence, the level of education, gender, and net income, reducing the ability of the model to predict the WTP. These findings necessitate further investigation and expanded research to include analysis of the effects of other potential factors, such as brewery size; reputation characteristics related to both beers and brewers; and label information, especially information related to health effects. However, these results open the way to a variety of strategies for valorizing non-alcoholic beer, from the promotion of its culture and beneficial effects (such as fewer calories and greater antioxidant power given the absence of alcohol, which could be useful for stimulating the interest of people with a high cultural level and young consumers) to the recognition of the greater intrinsic value of non-alcoholic beer with respect to the technological difficulty in obtaining qualitatively adequate products (which could also influence a greater willingness to pay, especially among people with higher incomes). An increase in the willingness to purchase/pay among different segments of the population could also be bolstered through differentiated packaging strategies (the use of eco-friendly materials and new formats), targeted advertising, and interaction with consumers on various social media platforms.

## Figures and Tables

**Table 1:** Distribution of total respondents (sample size = 392) by sociodemographic categories.

Characteristic	Category	Distribution of Respondents (%)
Number of Inhabitants in the Place of Residence E	<5000	8.4
	5001–20,000	17.4
	20,001–50,000	13.2
	50,001–200,000	51.0
	200,001–500,000	5.8
	>500,000	4.2
Italian Geographical Area ^1^	North-Western	3.7
	North-Eastern	4.7
	Central	2.6
	Southern	88.5
	Insular	0.5
Age Group	18–30	22.6
	31–40	12.1
	41–50	25.3
	51–60	32.1
	61–70	7.4
	71–80	0.5
	>80	0
Gender	Female	51.6
	Male	48.4
	Other	0
Education Level	Compulsory education (age 18+)	5.8
	High school diploma (age 18+)	21.6
	Bachelor’s degree	18.4
	Master’s degree	25.3
	Specialization/masters/PhD	28.9
Employment Situation	Student	18.9
	Unemployed	3.2
	Manager	12.1
	Employee	31.1
	Worker	4.7
	Teacher at nursery, primary, middle, or high schools	10.0
	University professor	9.5
	Retired	5.8
	Housewife	4.7
Annual Net Income (in EUR)	Less than 12,000	20.0
	From 12,001 to 18,000	12.6
	From 18,001 to 24,000	20.5
	From 24,001 to 30,000	16.8
	From 30,001 to 36,000	11.1
	From 36,001 to 50,000	10.0
	More than 50,000	9.0

^1^ Regions of Northwestern Italy: Lombardia, Piemonte, Valle d’Aosta, and Liguria; regions of Northeastern Italy: Veneto, Friuli-Venezia Giulia, Trentino-Alto Adige, and Emilia-Romagna; regions of Central Italy: Toscana, Umbria, Lazio, and Marche; regions of Southern Italy: Abruzzo, Molise, Puglia, Campania, Basilicata, and Calabria; regions of Insular Italy: Sicilia and Sardegna.

**Table 2:** Distribution of total respondents by consumption habits for alcoholic beer; knowledge and preference for non-alcoholic beers; and willingness to pay.

Question	Possible Answer	Distribution of Respondents (%)
Where do you consume alcoholic beer?	Mostly at home	22.1
	Mostly in pubs and beer houses	23.7
	Mostly in pizzerias	20.0
	Mostly in social clubs	0
	Wherever it happens	34.2
What type of alcoholic beer do you prefer to drink?	White	10.5
	Blond	64.7
	Red	19.5
	Dark	5.3
Have you ever drunk non-alcoholic beer?	Yes	63.2
	No	12.6
	No, but I’m curious about it and sooner or later, I’ll taste it	24.2
What are the reasons why you drink or would drink non-alcoholic beer?	For simple curiosity	35.8
	For a change	5.8
	To be able to drive without problems	31.0
	For health reasons	13.2
	To introduce fewer calories	14.2
What type of non-alcoholic beer do you prefer or think you would prefer to drink?	White	15.3
	Blond	66.3
	Red	14.7
	Dark	3.7
What type of packaging and format would you prefer for non-aocoholic beer?	33 cL glass bottle	73.2
	50 cL glass bottle	13.2
	75 cL glass bottle	2.6
	33 cL can	9.5
	50 cL can	1.5
In a public place you would prefer to consume non-alcoholic beer	On tap	56.3
	Bottled	43.7
Please indicate on the scale provided the price (in EUR/33 CL bottle) you think is fair to pay for the purchase of non-alcoholic beer.	Less than EUR 1.00	5.3
	Between EUR 1.00 and EUR 1.50	18.9
	Between EUR 1.51 and EUR 2.00	26.8
	Between EUR 2.00 and EUR 2.50	14.2
	Between EUR 2.51 and EUR 3.00	15.3
	Between EUR 3.01 and EUR 3.50	7.9
	Between EUR 3.51 and EUR 4.00	5.3
	Between EUR 4.01 and EUR 4.50	3.7
	Between EUR 4.51 and EUR 5.00	2.6

**Table 3:** Predictors of willingness to pay (WTP) for non-alcoholic beer.

Factors	Coefficients	Standard Error (SE)	*t*-Value	Significance (*p*)
Constant	0.278	1.415	0.196	0.846
Inhabitants of the place of residence	–0.213	0.281	–0.756	0.456
Italian geographical area ^1^	0.338	0.713	0.473	0.640
Age *	–0.726	0.373	–1.945	0.062
Gender	0.396	0.719	0.551	0.586
Education Level	–0.055	0.321	–0.172	0.865
Occupation	–0.074	0.160	–0.467	0.644
Annual net income	0.298	0.267	1.115	0.275
Place of beer consumption	0.153	0.235	0.651	0.520
Alcoholic beer preference	–0.719	0.502	–1.430	0.164
Practical knowledge of non-alcoholic beer	–0.272	0.365	–0.744	0.463
Reason to possibly drink non-alcoholic beer	–0.001	0.229	–0.039	0.969
Type of non-alcoholic beer (i.e., color of non-alcoholic beer) that the respondent prefers or would prefer to drink *	0.893	0.529	1.689	0.100
Type of preferred packaging and format for non-alcoholic beer	–0.159	0.528	–0.300	0.766
Preference for draft or bottled beer	–0.129	0.563	–0.228	0.821

^1^ Regions of Northwestern Italy: Lombardia, Piemonte, Valle d’Aosta, and Liguria; regions of Northeastern Italy: Veneto, Friuli-Venezia Giulia, Trentino-Alto Adige, and Emilia-Romagna; regions of Central Italy: Toscana, Umbria, Lazio, and Marche; regions of Southern Italy: Abruzzo, Molise, Puglia, Campania, Basilicata, and Calabria; regions of Insular Italy: Sicilia and Sardegna. *: Significant factors at *p* < 0.1

## Data Availability

The original contributions presented in the study are included in the article/Supplementary Material, further inquiries can be directed to the corresponding author.

## Supplementary Materials

The following supporting information can be downloaded at https://www.mdpi.com/article/10.3390/foods14132399/s1: Supplementary Table S1. The Italian-language version of the questionnaire can be viewed at the following link: https://docs.google.com/forms/d/e/1FAIpQLSe4rN17-fUVy_0uKBPX9nyEegvKWDRLrBWwJzbU9L1P3QeCew/viewform?usp=header.

## Abbreviations

The following abbreviations are used in this manuscript:

ABV	Alcohol by volume
WTP	Willingness to pay
NoLo	No- and low-alcohol beer
