# Supplementary material for: The Willingness to Pay for Non-Alcoholic Beer: A Survey on the Sociodemographic Factors and Consumption Behavior of Italian Consumers
Source: Foods. 2025 Jul 7;14(13):2399. doi: 10.3390/foods14132399 (PMC12248504; doi:10.3390/foods14132399)
Supplement: Supplementary file 1 [file foods-14-02399-s001.zip › foods-3686984-supplementary.pdf]

**Table S1.** Structure of the third section of the questionnaire.

| N. | Topic of the Questions | Possible answers |
|---|---|---|
| 1 | Where do you consume beer? | Mostly at home/Mostly in pubs and beer houses/Mostly in pizzerias/Mostly in a social club/Wherever it happens |
| 2 | What type of ALCOHOLIC beer do you prefer to drink? | White/Blond/Red/Dark |
| 3 | Have you ever drunk non-alcoholic beer? | Yes/No/ No, but I'm curious about it and sooner or later I'll taste it |
| 4 | What are the reasons why you drink or would drink NON-ALCOHOLIC beer? | For simple curiosity/For a change/To be able to drive without problems/For health reasons/To introduce fewer calories |
| 5 | What type of NON-ALCOHOLIC beer do you prefer or think you would prefer to drink? | White/Blond/Red/Dark |
| 6 | What type of packaging and format would you prefer for NON-ALCOHOLIC beer? | 33 cL glass bottle/50 cL glass bottle/75 cL glass bottle/33 cL can/50 cL can |
| 7 | In a public place you would prefer to consume NON-ALCOHOLIC beer: | On tap/Bottled |
| 8 | Please indicate on the scale provided what price (in Euro/33cl bottle) you think is fair to pay for the purchase of NON-ALCOHOLIC beer. | Less than € 1.00/Between € 1.00 and €1.50/Between € 1.51 and € 2.00/Between € 2.00 and € 2.50/Between € 2.51 and € 3.00/Between € 3.01 and € 3.50/Between € 3.51 and € 4.00/Between € 4.01 and € 4.50/Between € 4.51 and € 5.00 (ranges based on Italian market prices for non-alcoholic beers) |
